# Transmission of Smoking across Three Generations in Finland

**DOI:** 10.3390/ijerph13010074

**Published:** 2015-12-24

**Authors:** Salma E. T. El-Amin, Jaana M. Kinnunen, Hanna Ollila, Mika Helminen, Joana Alves, Pirjo Lindfors, Arja H. Rimpelä

**Affiliations:** 1 School of Health Sciences, University of Tampere, 33014 Tampere, Finland; salma.el.amin@staff.uta.fi (S.E.T.E.-A.); mika.helminen@uta.fi (M.H.); pirjo.lindfors@uta.fi (P.L.); arja.rimpela@uta.fi (A.H.R.); 2 Tobacco, Gambling and Addiction Unit, National Institute for Health and Welfare, 00271 Helsinki, Finland; hanna.ollila@thl.fi; 3 Science Centre, Pirkanmaa Hospital District, 33521 Tampere, Finland; 4 National School of Public Health, Lisbon NOVA University, P-1600-560 Lisbon, Portugal; joana.alves@ensp.unl.pt; 5 PERLA—Tampere Centre for Childhood, Youth and Family Research, University of Tampere, 33104 Tampere, Finland; 6 Department of Adolescent Psychiatry, Pitkäniemi Hospital, Tampere University Hospital, 33380 Nokia, Finland

**Keywords:** smoking, tobacco use, adolescents, intergenerational transmission, parents, grandparents

## Abstract

The influence of parents’ smoking on children’s smoking is well known, but few studies have examined the association between grandparents’ and grandchildren’s smoking. We studied the association between paternal and maternal grandparents’ smoking and their grandchildren’s tobacco use and assessed whether parents’ smoking is a mediator in this process. Data were obtained from a national survey of 12–18-year-old Finns in 2013 (*N* = 3535, response rate 38%). Logistic regression and mediation analyses were used. Both boys and girls had higher odds for smoking experimentation, daily smoking and other tobacco or tobacco-like product use if their mother, father or any of the four grandparents were current or former smokers. When parents’ and grandparents’ smoking status were included in the same model, grandparents’ smoking generally lost statistical significance. In the mediation analysis, 73% of the total effect of grandparents’ smoking on grandchildren’s daily smoking was mediated through parents’ smoking, 64% on smoking experimentation and 63% on other tobacco or tobacco-like product use. The indirect effect of a mother’s smoking was higher than that of a father’s. To conclude, paternal and maternal grandparents’ smoking increases grandchildren’s tobacco use. The influence is mainly, but not completely, mediated through parents’ smoking.

## 1. Introduction

Family has an important role in providing models of behaviors and shaping the tobacco practices, values, beliefs and attitudes of children and adolescents [1,2,3]. Children learn to construct their reality based on early experiences in their environment, peer behaviors and cultural norms [2,4]. The effect of parental smoking on children’s smoking has been well documented [5], but there have been differences found regarding whether a mother’s or father’s smoking is a more important risk factor and regarding whether the effect varies based on the child’s gender [6,7,8,9,10]. In their systematic review and meta-analysis, Leonardi-Bee *et al*. [5] concluded that having two smoking parents increases the child’s smoking risk compared to having just one smoking parent. They also concluded that a mother’s smoking is a slightly more prominent risk factor than a father’s smoking (OR 2.19 *vs*. OR 1.66). They also provided some evidence of a sex-specific effect in which boys were more substantially influenced by paternal smoking, while girls were influenced more by maternal smoking. 

Despite the vast evidence of the parental influence on smoking initiation, the influence of grandparents’ smoking and the intergenerational transmission of smoking behavior beyond two generations have rarely been studied [6,7]. However, the increased life expectancy of both men and women in industrialized countries [11] means that grandparents live longer and have more opportunities to witness their grandchildren’s development from early childhood to adolescence and even adulthood and thus their smoking may play a role in the initiation of smoking among their grandchildren. Escario and Wilkinson [12] showed that smoking by at least one grandparent living in the same family as grandchildren increases the odds of smoking for boys but not for girls. A study from the U.S. examined the smoking behavior across three generations and showed that smoking behaviors are transferred from preceding generations to later generations and that parents have a mediating role in this process [8]. There are no published studies in which the effects of maternal and paternal grandparents’ smoking on grandchildren’s smoking have been studied separately; nor have there been studies on the use of tobacco or tobacco-like products other than cigarettes. In this study, other tobacco or tobacco-like products include snus, water pipes and electronic cigarettes, and the shorter term “other tobacco products” will be used to encompass these products, although electronic cigarettes do not actually contain tobacco.

In this study, we addressed the transmission of smoking across three generations. The research questions were as follows:

(1) Are maternal and paternal grandmothers’ and grandfathers’ smoking related to their grandchildren’s smoking and use of other tobacco products?
(2) Is the influence of grandparents’ smoking independent of parents’ smoking or is it mediated through parental smoking behavior?

In addition, we studied the relationship of mother’s and father’s smoking to their children’s smoking and use of other tobacco products as well as differences between genders. 

## 2. Methods

### 2.1. Sampling and Participants 

Data from the 2013 Adolescent Health and Lifestyle Survey (AHLS), the nationwide monitoring system on adolescent health and health behaviors in Finland was used. AHLS is a cross-sectional postal survey, with an option to answer securely online. It has been conducted biennially since 1977. A nationally representative sample of individuals aged 12, 14, 16 and 18 years was obtained from the national Population Register Centre. All Finns born on sample days in June, July or August in each age group were selected to minimize the age variation within age groups. The Ethics Committee of the Tampere region approved the study protocol. Filling in the questionnaire was considered an adolescent’s consent to participate, and no parental consent was needed according to the Ethics Committee. In January 2013, self-administered questionnaires in Finnish were sent to 9398 adolescents. They were followed by three reminders to non-responders. The number of responders to the questionnaire was 3535, indicating a response rate of 38% (including 1405 boys and 2130 girls). 

### 2.2. Measures

#### 2.2.1. Children’s Smoking

Children’s smoking status was assessed with the questions: “Have you ever tried smoking?” with the options “No” and “Yes”; “How many cigarettes have you smoked during your life-time until now?” with the options “None”, “Only one”, “Approximately 2–50” and “More than 50”; and “Which of the following options best describes your current smoking?” with the options “I smoke once per day or more often”, “I smoke once per week or more often but not daily”, “I smoke less than once per week”, “I have stopped smoking” and “I don’t smoke”. Based on the answers, the smoking status was divided into three groups: Never smokers (never tried smoking), experimenters (tried but did not smoke daily) and daily smokers (reported daily smoking and had smoked >50 cigarettes during their lifetimes). For the analyses, dichotomization was conducted for tried smoking (experimenting) and daily smoking (No/Yes) based on the smoking initiation process where “tried smoking” represents the first steps and “daily smoking” represents regular smoking where nicotine addiction has a stronger role (See Table 1).

**Table 1:** Distribution of children’s smoking experimentation, daily smoking and other tobacco product use * and parents’ and grandparents’ smoking by gender.

Smoking	Girls	Boys	All
	(*n* = 2130)	(*n* = 1405)	(*N* = 3535)
	% (*n*)	% (*n*)	% (*n*)
Children			
*Tried smoking*			
No	59.8 (1267)	61.6 (852)	60.5 (2120)
Yes	40.2 (851)	38.4 (531)	39.5 (1382)
*Daily smoking*			
No	89.4 (1901)	91.0 (1277)	90.0 (3178)
Yes	10.6 (226)	9.0 (126)	10.0 (352)
*Other tobacco product use* *			
No	72.6 (1547)	67.2 (939)	70.5 (2486)
Yes	27.4 (583)	32.8 (458)	29.5 (1041)
Parents			
*Father’s smoking status*			
Never smoker	56.6 (1153)	56.1 (749)	56.4 (1902)
Ex-smoker	19.9 (406)	22.7 (303)	21.0 (705)
Current smoker	23.4 (477)	21.1 (282)	22.5 (759)
*Mother’s smoking status*			
Never smoker	70.0 (1451)	71.7 (962)	70.7 (2413)
Ex-smoker	13.9 (288)	14.8 (199)	14.3 (487)
Current smoker	16.1 (333)	13.5 (181)	15.1 (514)
Paternal grandparents			
*Grandfather’s smoking status*			
Never smoker	75.5 (1070)	73.2 (736)	74.5 (1806)
Ex-smoker	14.4 (204)	17.0 (171)	15.5 (375)
Current smoker	10.2 (144)	9.8 (99)	10.0 (243)
*Grandmother’s smoking status*			
Never smoker	83.7 (1427)	85.8 (975)	84.5 (2402)
Ex-smoker	7.8 (133)	7.2 (82)	7.6 (215)
Current smoker	8.5 (145)	7.0 (80)	7.9 (225)
Maternal grandparents			
*Grandfather’s smoking status*			
Never smoker	71.0 (1132)	71.4 (761)	71.1 (1893)
Ex-smoker	17.1 (273)	17.6 (188)	17.3 (461)
Current smoker	11.9 (190)	11.0 (117)	11.5 (307)
*Grandmother’s smoking status*			
Never smoker	81.3 (1484)	82.9 (979)	81.9 (2463)
Ex-smoker	8.5 (155)	8.6 (102)	8.5 (257)
Current smoker	10.2 (186)	8.5 (100)	9.5 (286)

* Snus, water pipe and electronic cigarettes.

The use of other tobacco or tobacco-like products was assessed with the following questions: “Have you ever tried snus?” with the options “Have not tried”, “Tried once”, “Have used 2–50 times” and “Have used more than 50 times”; and “Have you ever tried water pipe?” and “Have you ever tried electronic cigarettes?” with the options for both questions “I don’t know what this is”, “No”, “I have tried this product once or twice”, “I have tried this product 20 times or fewer” and “I have tried this product more than 20 times”. For the analyses, these three questions were combined as one dichotomized variable “other tobacco product use” such that reporting a trial of at least one of these products was coded as “Yes”, and otherwise the response was coded as “No”.

#### 2.2.2. Parents’ and Grandparents’ Smoking

Data on parents’ and grandparents’ smoking were assessed with the questions “Have your parents smoked during your lifetime?” and “Have your grandparents smoked during your lifetime?” separately for father and mother, paternal grandfather and grandmother, and maternal grandfather and grandmother. The options were “Never”, “Has stopped”, “Smokes currently”, and “I don’t have one or I don’t know”. In the analyses, the answers for the option “I don’t have one or I don’t know” were included in the category “Never”. The proportion of missing values was 2.8% for father, 1.5% for mother, 28.9% for paternal grandfather, 16.6% for paternal grandmother, 22.3% for maternal grandfather, and 12.4% for maternal grandmother. Additionally, a combined variable was created for parents’, paternal grandparents’ and maternal grandparents’ smoking with categories “Neither of them smokes”, “One or both has stopped”, “One of them smokes” and “Both of them smoke”. 

#### 2.2.3. Other Variables

Parents’ employment status was assessed and categorized separately for fathers and mothers as “Working”, “Unemployed” and “Other” (retired or on a long sick leave). Parents’ education was assessed separately and combined into one variable according to the highest level that the parents had achieved with the categories “high” (more than 12 years of education), “middle” (9–12 years) and “low” (9 years or fewer).

### 2.3. Analysis of Non-Response

Boys were underrepresented among the respondents (40.6%) compared with the overall sample (50.7%). For age, adolescents aged 12 years were overrepresented (16.3% *vs*. 13.5%), and those aged 18 years were underrepresented (25.7% and 30.4%, respectively), while differences were small for those aged 14 years and 16 years. Boys were more likely to be non-responders (*p* = 0.01), but the differences between age groups were not significant (*p* = 0.216). The impact of non-response on the reports of parents’ and grandparents’ smoking was assessed by dividing the responders into four groups according to how promptly they had answered the survey. It was assumed that the later the person answered, the more he/she resembled a non-responder. There were no systematic differences between the groups that had answered early or late that would have suggested an over- or underrepresentation of parental or grandparental smoking among the non-respondents (Appendix Table A1).

### 2.4. Data Analysis

Logistic regression analysis was used to study the association of parents’ and grandparents’ smoking with children’s smoking and other tobacco product use. Results are presented as odds ratios (ORs) and 95% confidence intervals (CIs). First, age-adjusted ORs and 95% CIs for boys’ and girls’ tobacco use variables were calculated according to the mother’s, father’s, and each of the four grandparent’s smoking statuses (Table 2). Second, ORs and 95% CIs were calculated for children’s tobacco use variables according to each grandparent’s smoking status, adjusting first for age and sex (Model 1, Table 3) and then for parents’ smoking status, employment status and education (Model 2, Table 3). Third, logistic regression analysis was conducted for children’s tobacco use according to the number of smokers among parents, maternal grandparents and paternal grandparents (Model 1, Table 4), adjusting for age and sex. Finally, all three variables were included in the analysis at the same time, adjusting for age, sex, parents’ education, and employment status (Model 2, Table 4). 

The Pearson χ^2^ test was used to examine the statistical significance; *p*-values in Table 2, Table 3 and Table 4 represent the statistical significance of the relationship between children’s tobacco use and parents’/grandparents’ smoking variables (Wald test). The logistic regression analyses were conducted with IBM SPSS Statistics v. 20.0 software (IBM Inc. Armonk, NY, USA).

**Figure 1:**
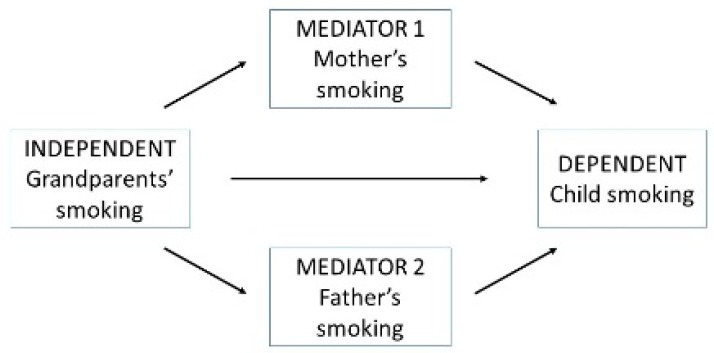
The relationships between grandparents’ smoking, mother’s and father’s smoking and child’s smoking.

The mediation analysis [13] was performed to assess how much of the effect of the exposure to grandparents’ smoking on children’s smoking is mediated through mothers’ and fathers’ smoking. Grandparents’ smoking was assumed to have an effect on both mothers’ and fathers’ smoking and also on children’s smoking (Figure 1). Mothers’ and fathers’ smoking was assumed to have effect only on children’s smoking. All the dependent, independent and mediator variables were coded as binary (No = 0, Yes = 1). For the mother and father, the categorization was 0 = Never smoker and 1 = Smoker or ex-smoker, and for grandparents, 0 = All grandparents never smokers and 1 = One or more grandparents smokers or ex-smokers. STATA (version 13.1) software with a “binary-mediation” program was used for mediation analyses, together with a “bootstrap” command for producing the confidence intervals [14]. 

## 3. Results

### 3.1. Smoking among Children, Their Parents and Grandparents

Overall, 39.5% of the 12–18-year-old respondents had tried smoking, and 10.0% of them smoked daily (Table 1). Smoking, both experimentation and daily smoking, was somewhat more prevalent among girls, but the difference was not significant. Of all respondents, 29.5% had tried other tobacco products, boys more often than girls (*p* < 0.001). In total, 15.1% of mothers and 22.5% of fathers smoked currently, compared to less than 12% of all grandparents.

### 3.2. Association of Parents’ and Grandparents’ Smoking Statuses with Children’s Tobacco Use

When analyzing boys and girls separately and adjusting for age, fathers’ and mothers’ current smoking was associated with children’s smoking experimentation and daily smoking as well as with other tobacco product use (Table 2). The associations were somewhat stronger among girls; the strongest association was found between mothers’ current smoking and girls’ daily smoking. Additionally, fathers’ and mothers’ former smoking were associated with the children’s smoking experimentation and daily smoking as well as with other tobacco product use (See also Appendix Table A2).

**Table 2:** Age-adjusted ORs and 95% CIs for boys’ and girls’ smoking experimentation, daily smoking and other tobacco product use * by parents’ and grandparents’ smoking statuses.

Smoking Status of Parents and Grandparents	Tried Smoking		Daily Smoking		Other Tobacco Product Use *	
	Girls	Boys	Girls	Boys	Girls	Boys
	OR (95% CI)	OR (95% CI)	OR (95% CI)	OR (95% CI)	OR (95% CI)	OR (95% CI)
Parents						
*Father’s smoking status*						
Never smoker	1.00	1.00	1.00	1.00	1.00	1.00
Ex-smoker	**1.73** (1.35–2.22)	**1.92** (1.44–2.57)	**2.27** (1.56–3.30)	**2.64** (1.66–4.21)	**1.45** (1.11–1.90)	**1.53** (1.14–2.05)
Current smoker	**2.14** (1.70–2.71)	**1.90** (1.41–2.55)	**3.16** (2.25–4.44)	**2.32** (1.43–3.76)	**1.99** (1.55–2.54)	**1.50** (1.11–2.03)
*p*-value	˂0.001	˂0.001	˂0.001	˂0.001	˂0.001	0.009
*Mother’s smoking status*						
Never smoker	1.00	1.00	1.00	1.00	1.00	1.00
Ex-smoker	**1.88** (1.43–2.48)	**2.06** (1.49–2.86)	**2.33** (1.55–3.50)	1.55 (0.90–2.66)	**1.96** (1.46–2.62)	**1.44** (1.03–2.01)
Current smoker	**2.71** (2.08–3.52)	**1.56** (1.11–2.18)	**5.18** (3.72–7.22)	**2.91** (1.81–4.68)	**2.85** (2.18–3.72)	**1.46** (1.04–2.05)
*p*-value	˂0.001	˂0.001	˂0.001	˂0.001	˂0.001	0.010
Paternal grandparents						
*Grandfather’s smoking status*						
Never smoker	1.00	1.00	1.00	1.00	1.00	1.00
Ex-smoker	**1.42** (1.03–1.97)	1.36 (0.96–1.94)	1.29 (0.79–2.09)	**2.19** (1.24–3.85)	**1.44** (1.02–2.03)	1.35 (0.94–1.93)
Current smoker	**1.84** (1.26–2.70)	1.34 (0.90–2.18)	**2.63** (1.59–4.33)	1.74 (0.85–3.57)	**2.40** (1.61–3.57)	1.17 (0.74–1.85)
*p*-value	0.005	0.210	0.002	0.006	˂0.001	0.291
*Grandmother’s smoking status*						
Never smoker	1.00	1.00	1.00	1.00	1.00	1.00
Ex-smoker	**2.47** (1.67–3.64)	0.71 (0.43–1.17)	**1.93** (1.14–3.25)	1.23 (0.53–2.82)	**1.92** (1.28–2.86)	0.76 (0.45–1.28)
Current smoker	1.25 (0.86–1.83)	**1.73** (1.06–2.81)	**1.71** (1.01–2.90)	**3.02** (1.50–6.09)	1.38 (0.92–2.06)	**1.84** (1.14–2.99)
*p*-value	˂0.001	0.055	0.005	0.001	0.005	0.054
Maternal grandparents						
*Grandfather’s smoking status*						
Never smoker	1.00	1.00	1.00	1.00	1.00	1.00
Ex-smoker	1.26 (0.95–1.68)	**1.72** (1.23–2.42)	1.11 (0.71–1.73)	**2.41** (1.42–4.08)	1.21 (0.89–1.65)	**1.48** (1.05–2.08)
Current smoker	**1.41** (1.00–1.97)	**1.84** (1.22–2.78)	1.31 (0.78–2.20)	**1.99** (1.02–3.89)	1.23 (0.85–1.79)	1.28 (0.84–1.96)
*p*-value	0.004	0.001	0.111	0.004	0.219	0.112
*Grandmother’s smoking status*						
Never smoker	1.00	1.00	1.00	1.00	1.00	1.00
Ex-smoker	**1.58** (1.10–2.27)	1.03 (0.67–1.59)	1.47 (0.85–2.53)	1.60 (0.84–3.06)	**2.07** (1.42–3.03)	1.18 (0.76–1.82)
Current smoker	**1.66** (1.19–2.32)	1.47 (0.95–2.29)	**2.86** (1.88–4.36)	**2.03** (1.00–4.10)	**1.66** (1.16–2.36)	0.97 (0.60–1.55)
*p*-value	˂0.001	0.205	˂0.001	0.078	˂0.001	0.895

* Snus, water pipe and electronic cigarettes; Note: OR is given in boldface when it indicates a statistically significant (*p* < 0.05) difference from the odds of the reference category.

**Table 3:** Adjusted ORs and 95% CIs for children’s smoking experimentation, daily smoking and other tobacco product use * by grandparents’ smoking status in two models ^†^.

Grandparents’ Smoking Status	Tried Smoking		Daily Smoking		Other Tobacco Product Use *	
	Model 1	Model 2	Model 1	Model 2	Model 1	Model 2
	OR (95% CI)	OR (95% CI)	OR (95% CI)	OR (95% CI)	OR (95% CI)	OR (95% CI)
**Paternal grandparents**						
*Grandfather’s smoking status*						
Never smoker	1.00	1.00	1.00	1.00	1.00	1.00
Ex-smoker	**1.40** (1.10–1.78)	1.16 (0.91–1.49)	**1.59** (1.10–2.28)	1.20 (0.82–1.76)	**1.40** (1.09–1.79)	1.21 (0.94–1.57)
Current smoker	**1.61** (1.20–2.14)	1.23 (0.91–1.66)	**2.27** (1.51–3.41)	1.50 (0.97–2.31)	**1.69** (1.25–2.28)	1.35 (0.99–1.84)
*p*-value	˂0.001	0.072	˂0.001	0.189	˂0.001	0.035
*Grandmother’s smoking status*						
Never smoker	1.00	1.00	1.00	1.00	1.00	1.00
Ex-smoker	**1.52** (1.13–2.05)	1.14 (0.84–1.56)	**1.68** (1.08–2.61)	1.10 (0.69–1.75)	1.32 (0.97–1.81)	1.03 (0.75–1.43)
Current smoker	**1.40** (1.04–1.89)	1.07 (0.79–1.46)	**2.07** (1.36–3.15)	1.34 (0.86–2.09)	**1.54** (1.13–2.09)	1.20 (0.87–1.66)
*p*-value	˂0.001	0.842	˂0.001	0.441	0.007	0.515
**Maternal grandparents**						
*Grandfather’s smoking status*						
Never smoker	1.00	1.00	1.00	1.00	1.00	1.00
Ex-smoker	**1.44** (1.16–1.80)	1.21 (0.97–1.51)	**1.49** (1.07–2.09)	1.14 (0.80–1.62)	**1.33** (1.06–1.67)	1.13 (0.89–1.43)
Current smoker	**1.56** (1.20–2.02)	1.26 (0.96–1.65)	**1.53** (1.02–2.30)	0.99 (0.64–1.53)	1.24 (0.94–1.64)	0.99 (0.74–1.33)
*p*-value	˂0.001	0.203	0.019	0.831	0.052	0.529
*Grandmother’s smoking status*						
Never smoker	1.00	1.00	1.00	1.00	1.00	1.00
Ex-smoker	1.30 (0.99–1.72)	1.01 (0.76–1.35)	**1.54** (1.02–2.34)	1.03 (0.66–1.59)	**1.57** (1.18–2.04)	1.26 (0.94–1.69)
Current smoker	**1.58** (1.21–2.06)	1.14 (0.86–1.51)	**2.60** (1.81–3.73)	**1.47** (1.00–2.17)	**1.35** (1.02–1.79)	0.99 (0.73–1.33)
*p*-value	˂0.001	0.383	˂0.001	0.260	0.002	0.163

* Snus, water pipe and electronic cigarettes; ^†^ Model 1: adjusted for age and sex; Model 2: parents’ and grandparents’ smoking simultaneously in the model, adjusted for age, sex and parents’ education and employment status; Note: OR is given in boldface when it indicates a statistically significant (*p* < 0.05) difference from the odds of the reference category.

**Table 4:** Adjusted ORs and 95% CIs for children’s smoking experimentation, daily smoking and other tobacco product use * by number of smoking parents and grandparents in two models ^†^.

Smoking Status of Parents and Grandparents	Tried Smoking		Daily Smoking		Other Tobacco Product Use *	
	Model 1	Model 2	Model 1	Model 2	Model 1	Model 2
	OR (95% CI)	OR (95% CI)	OR (95% CI)	OR (95% CI)	OR (95% CI)	OR (95% CI)
**Parents’ smoking**						
Both never smokers	1.00	1.00	1.00	1.00	1.00	1.00
One or both ex-smokers	**2.04** (1.71–2.42)	**1.93** (1.57–2.38)	**2.79** (2.11–3.69)	**2.77** (1.96–3.91)	**1.73** (1.45–2.07)	**1.70** (1.37–2.19)
One smoker	**1.87** (1.50–2.33)	**1.97** (1.50–2.58)	**2.20** (1.54–3.15)	**2.36** (1.52–3.68)	**1.78** (1.42–2.24)	**2.02** (1.52–2.67)
Both smokers	**2.55** (1.93–3.37)	**2.60** (1.82–3.71)	**5.63** (3.93–8.07)	**5.65** (3.55–9.02)	**2.42** (1.82–3.20)	**2.51** (1.74–3.63)
*p*-value	˂0.001	˂0.001	˂0.001	˂0.001	˂0.001	˂0.001
**Paternal grandparents’ smoking**						
Both never smokers	1.00	1.00	1.00	1.00	1.00	1.00
One or both ex-smokers	**1.42** (1.15–1.75)	1.04 (0.82–1.32)	**1.76** (1.28–2.41)	1.17 (0.81–1.71)	**1.35** (1.08–1.68)	0.98 (0.76–1.27)
One smoker	1.22 (0.93–1.60)	1.10 (0.81–1.49)	**1.85** (1.24–2.78)	1.50 (0.95–2.38)	1.28 (0.97–1.70)	1.16 (0.84–1.60)
Both smokers	**2.92** (1.65–5.15)	**1.97** (1.05–3.68)	**3.45** (1.73–6.86)	2.05 (0.95–4.42)	**3.16** (1.80–5.55)	**2.34** (1.42–4.91)
*p*-value	˂0.001	0.198	˂0.001	0.129	˂0.001	0.018
**Maternal grandparents’ smoking**						
Both never smokers	1.00	1.00	1.00	1.00	1.00	1.00
One or both ex-smokers	**1.34** (1.10–1.64)	1.19 (0.95–1.49)	**1.57** (1.14–2.14)	1.21 (0.85–1.73)	**1.38** (1.12–1.70)	1.18 (0.93–1.50)
One smoker	**1.50** (1.18–1.92)	1.67 (0.88–1.55)	**2.02** (1.40–2.91)	1.05 (0.67–1.66)	1.28 (0.99–1.67)	0.96 (0.70–1.30)
Both smokers	**2.00** (1.22–3.23)	1.58 (0.92–2.71)	**2.67** (1.33–5.33)	1.40 (0.64–3.04)	1.50 (0.88–2.58)	1.07 (0.59–1.95)
*p*-value	˂0.001	0.185	˂0.001	0.656	0.008	0.524

* Snus, water pipe and electronic cigarettes; ^†^ Model 1: adjusted for age and sex; Model 2: parents’ and grandparents’ smoking statuses included simultaneously in the model, adjusted for age, sex and parents’ education and employment status; Note: OR is given in boldface when it indicates a statistically significant (*p* < 0.05) difference from the odds of the reference category.

Grandparents’ current smoking and former smoking were related to the grandchildren’s smoking experimentation, daily smoking and other tobacco product use; however, the associations were not as strong as for parents’ smoking (Table 2). Some of these relationships were not significant (e.g., boys’ other tobacco product use with maternal and paternal grandfather and maternal grandmother; girls’ daily smoking and other tobacco product use with maternal grandfather). The strongest association was found between paternal grandmothers’ current smoking and boys’ daily smoking (3.02; 1.50 to 6.09).

As shown in Table 3, the grandparents’ current and former smoking statuses were associated with the grandchildren’s smoking experimentation, daily smoking and other tobacco product use in the age- and sex-adjusted Model 1. Most associations were significant, excluding maternal grandmother’s former smoking with smoking experimentation and paternal grandmothers’ former smoking and maternal grandfathers’ current smoking with other tobacco product use. When adjusting for parents’ smoking, employment status and education (Table 3, Model 2), the associations remained but were weakened, and significance was lost because of the strong association of parents’ smoking with grandparents’ and children’s smoking.

Children with two smoking parents or two smoking paternal or maternal grandparents had higher odds for smoking experimentation, daily smoking and other tobacco product use compared to when only one was a smoker or when parents or grandparents were former smokers (Table 4, Model 1). With all the variables from Model 1 along with the parents’ employment status and education in Model 2, the associations with grandparents’ smoking lost significance, with the exceptions of the relationships between two paternal grandparents’ current smoking and the grandchildren’s smoking experimentation and other tobacco product use.

### 3.3. Mediation Analysis 

A mediation analysis was used to test whether the effect of grandparents’ smoking on the grandchildren’s smoking was mediated through mothers’ and fathers’ smoking (Figure 1). The total effect is a summary of the direct effect (grandparents’ smoking) and the indirect effects (mother’s and father’s smoking) and it can be seen as a correlational measure between grandparents’ smoking and grandchildren’s smoking. Only about 10% (0.097) of grandchildren’s smoking experimentation, about 14% (0.137) of daily smoking and about 9% (0.086) of other tobacco product use is explained by the transmission of smoking from the grandparents to the grandchildren (Table 5). The total effect of grandparents’ smoking was mainly mediated through mothers’ and fathers’ smoking (indirect effects) for all three indicators of children’s smoking. The proportion of the total effect that is mediating through parents’ smoking was calculated by dividing the sum of the indirect effect by the total effect. Of the total effect of grandparents’ smoking, 64% of smoking experimentation, 73% of daily smoking, and 63% for other tobacco product use was mediated through parents’ smoking; and approximately one third (36%, 27% and 37%) of the total effect of grandparents’ smoking on grandchildren’s tobacco use was direct.

**Table 5:** Coefficients and their confidence intervals from mediation analysis of the association between grandparents’ smoking and grandchildren’s smoking experimentation, daily smoking and other tobacco product use *, considering mother’s and father’s smoking as mediators.

Effect	Tried Smoking	Daily Smoking	Other Tobacco Product Use *
**Indirect Effects**			
*Mother’s smoking*	0.036 (0.021–0.049)	0.066 (0.042–0.087)	0.041 (0.02–0.055)
*Father’s smoking*	0.026 (0.012–0.038)	0.034 (0.015–0.053)	0.013 (0.00–0.027)
**Direct effect**			
*Grandparents’ smoking*	0.035 (−0.015–0.073)	0.037 (−0.03–0.111)	0.032 (−0.01–0.074)
**Total effect**	0.097 (0.049–0.135)	0.137 (0.07–0.209)	0.086 (0.04–0.125)
Proportion of the total effects mediated through parents	64%	73%	63%
Proportion of the total effect that is direct from grandparents to grandchildren	36%	27%	37%

* Snus, water pipe and electronic cigarettes.

## 4. Discussion

The results of this study demonstrate the association between grandparents’ smoking and their grandchildren’s tobacco use. The association was noted for both paternal and maternal grandparents and for experimentation, daily smoking and use of other tobacco or tobacco-like products. The transmission of smoking from grandparents to grandchildren was mainly mediated through mother’s and father’s smoking, which were also strongly related to children’s tobacco use. One-third of the effect from grandparents was direct, not mediated through the parents. Children having two smoking parents or paternal or maternal grandparents were more likely to smoke, as were those with formerly smoking parents or grandparents.

The association between children’s and their grandparents’ smoking and the mediation effect of parents’ smoking are consistent with the study by Vandewater *et al.* [8], which is the only report studying three generations and all grandparents and not only those who live in the same family with their grandchildren. Our study adds to the previous knowledge by showing that there seems to be a direct effect of grandparents on adolescent smoking behavior and that both the paternal and maternal grandparents are important. 

A number of mechanisms, both social, psychological, and genetic, have been proposed to explain the influence of parents’ smoking on their children’s smoking. These mechanisms are likely to be valid, at least partly, in explaining the influence of grandparents’ smoking behavior as well. The social and psychological mechanisms include direct modeling of behavior, in which parents and grandparents serve as role models, the transmission of norms and attitudes towards smoking, and (grand)parenting styles like a home smoking ban and controlling access to tobacco products and to certain friendship networks [15,16]. Genetics and biological pathways have also been shown to have a role in smoking behavior and nicotine addiction [17,18] and the role of second-hand smoke should not be forgotten [19]. Both genetic and environmental factors can explain smoking initiation and quantities of cigarettes smoked and environmental factors can regulate the expression of genetic predisposition [18,20]. Interestingly, non-biological stepparents’ smoking has been shown to influence adolescents’ smoking as significantly as parents’ smoking [21], supporting the important role of social and environmental factors. 

With the increasing life expectancy, children have more possibilities to spend time with their grandparents, explaining why their influence on different aspects of children’s life is likely to be higher than in previous decades. In Finland, where this study was conducted, 30% of 12-year-old children had all four grandparents alive in 2011 [22]. A majority of grandparents actively provide childcare for their grandchildren in Finland [23]. The evidence also suggests that the intergenerational influences and transmission of values, attitudes, and patterns of behavior between grandchildren and grandparents today are strong, despite changes in the society as a whole and in terms of family structure and socioeconomic context [24,25,26]. Grandparents’ possible role in adolescent smoking prevention programs is worth studying because family interventions have been shown to have positive effects [27].

Finland is an interesting context to study the effect of family smoking because of its strict smoking prevention legislation [28]. All advertisement and sales promotion are forbidden, sales of tobacco products to minors under the age of 18 is forbidden, tobacco products are not displayed in retail sales, and smoking is not allowed in public places, restaurants, cafés, workplaces, schools and in places which minors can access. This means that the exposure within the family may be more influential in a Finnish society than in a society where smoking and tobacco products are easily seen and accessed by minors, and where tobacco industry can advertise their products. Cross-country comparisons could bring valuable insights into the mechanisms of the intergenerational transmission of smoking and into the actions needed to prevent it. In countries with strong tobacco control policies, family smoking could be one of the remaining issues to tackle and mostly with other means than legislative bans and restrictions (e.g., family interventions and smoking cessation support in health care). Countries with less comprehensive tobacco control policies could benefit from stronger implementation of the Framework Convention on Tobacco Control (FCTC) as the first-stage prevention.

Some limitations of our study should be noted. The data are based on adolescents’ self-reports, and we cannot know how accurately they have reported their grandparents’ smoking. Conversely, adolescents’ reports reflect their perceptions of their grandparents’ smoking, which can be considered even more important than the actual grandparent’s smoking status. We also do not know what close contact, if any, the children had with their grandparents; this information would have provided more insight into the role of grandparents and may have modified the observed effects. When children or grandchildren are asked about grandparents’/parents’ smoking, the exposure to environmental tobacco smoke during early childhood may be omitted, which may dilute the effects in our study. We did not have information on the age of parents or grandparents which may have been relevant because smoking has diminished in these age groups. On the other hand, we have shown that the strength of the association between the parents’ and child’s smoking did not change over a period of three decades [29] which is why the lack of age information hardly produces any bias. Important factors in the initiation and continuation of smoking are siblings’ and peers’ smoking [5] which were not available in our data. The low response rate may alter the generalizability of this study, although the indirect non-response analysis did not suggest any bias in the adolescents’ reports of their grandparents’ smoking.

## 5. Conclusions

Paternal and maternal grandparents’ smoking is associated with grandchildren’s tobacco use. The influence is mainly, but not completely, mediated through parents’ smoking, suggesting an independent role of grandparents in smoking initiation. The role of grandparents in the prevention of adolescent smoking is worth considering in future prevention programs. Implementation of strong tobacco control policies is essential in order to reduce smoking among adults and the elderly population—which are the sources of the intergenerational transmission of smoking. Understanding the relationships between children and their grandparents’ smoking statuses in different settings as well as the type and quality of contacts between children and their grandparents would help us to understand the role of grandparents more thoroughly. 

## Appendix

**Table A1:** Distribution of children’s smoking experimentation, daily smoking and other tobacco product use ***** and parents’ and grandparents’ smoking by mailing order and the *p*-value.

Smoking	Round 1, %	Round 2, %	Round 3, %	Round 4, %	*p*-Value
Parents					
*Father’s smoking status*					0.289
Never smoker	57.7	57.3	53.5	53.2	
Ex-smoker	19.8	22.4	22.5	22.4	
Current smoker	22.5	20.3	24.0	24.4	
*Mother’s smoking status*					0.869
Never smoker	70.4	71.5	71.0	70.2	
Ex-smoker	14.9	13.3	13.9	12.8	
Current smoker	14.7	15.1	15.0	17.0	
Paternal grandparents					
*Grandfather’s smoking status*					0.198
Never smoker	73.4	74.3	78.7	72.9	
Ex-smoker	15.6	17.5	12.7	16.9	
Current smoker	11.0	8.1	8.7	10.1	
*Grandmother’s smoking status*					0.341
Never smoker	83.2	87.5	85.5	85.1	
Ex-smoker	8.4	5.3	7.2	7.6	
Current smoker	8.4	7.2	7.4	7.2	
Maternal grandparents					
*Grandfather’s smoking status*					0.008
Never smoker	72.2	66.5	75.6	64.3	
Ex-smoker	16.7	21.3	14.4	19.8	
Current smoker	11.2	12.3	10.0	15.9	
*Grandmother’s smoking status*					0.596
Never smoker	82.8	80.3	82.0	79.8	
Ex-smoker	7.7	9.6	9.4	10.1	
Current smoker	9.6	10.1	8.5	10.1	

***** Snus, water pipe and electronic cigarettes.

**Table A2:** ORs and 95% CIs for boys’ and girls’ smoking experimentation, daily smoking and other tobacco product use * by parents’ smoking statuses, adjusted for age, parents’ education and employment status.

Smoking Status of Parents and Grandparents	Tried Smoking		Daily Smoking		Other Tobacco Product Use *	
	Girls	Boys	Girls	Boys	Girls	Boys
	OR (95% CI)	OR (95% CI)	OR (95% CI)	OR (95% CI)	OR (95% CI)	OR (95% CI)
**Parents**						
*Father’s smoking status*						
Never smoker	1.00	1.00	1.00	1.00	1.00	1.00
Ex-smoker	**1.60** (1.23–2.07)	**1.88** (1.39–2.55)	**1.86** (1.26−2.77)	**2.39** (1.45−3.93)	**1.51** (1.14−1.99)	**1.41** (1.04−1.91)
Current smoker	**1.99** (1.55–2.56)	**1.89** (1.38–2.58)	**2.61** (1.83−3.73)	**2.11** (1.26−3.54)	**1.92** (1.48−2.50)	**1.41** (1.02−1.94)
*p*-value	˂0.001	˂0.001	˂0.001	0.001	˂0.001	0.031
*Mother’s smoking status*						
Never smoker	1.00	1.00	1.00	1.00	1.00	1.00
Ex-smoker	**1.86** (1.40–2.48)	**1.84** (1.30–2.59)	**2.30** (1.51−3.49)	1.17 (0.65−2.11)	**2.11** (1.56−2.86)	1.41 (0.99−2.00)
Current smoker	**2.44** (1.84−3.23)	1.41 (0.98−2.03)	**4.50** (3.15−6.44)	**2.30** (1.36−3.88)	**2.80** (2.10−3.73)	1.39 (0.96−2.00)
*p*-value	˂0.001	0.001	˂0.001	0.007	˂0.001	0.067

***** Snus, water pipe and electronic cigarettes; Note: OR is given in boldface when it indicates a statistically significant (*p* < 0.05) difference from the odds of the reference category.
